# Burning mouth syndrome: A diagnostic and therapeutic dilemma

**DOI:** 10.4317/jced.50764

**Published:** 2012-07-01

**Authors:** Ashish Aggarwal, Sunil R. Panat

**Affiliations:** 1 MDS, Senior Lecturer. Department of Oral Medicine and Radiology, Institute of Dental Sciences, Bareilly (U.P), India.; 2 MDS, Principal, Professor and Head. Department of Oral Medicine and Radiology, Institute of Dental Sciences, Bareilly (U.P), India.

## Abstract

Burning mouth syndrome (BMS) has been considered an enigmatic condition because the intensity of pain rarely corresponds to the clinical signs of the disease. Various local, systemic and psychological factors are associated with BMS, but its etiology is not fully understood. Also there is no consensus on the diagnosis and classification of BMS. A substantial volume of research has been focused on BMS during the last two decades. Progress has been made but the condition remains a fascinating, yet poorly understood area, in the field of oral medicine. Recently, there has been a resurgence of interest in this disorder with the discovery that the pain of BMS may be neuropathic in origin and originate both centrally and peripherally. The aim of this paper is to explore the condition of BMS with the specific outcome of increasing awareness of the condition.

** Key words:**Burning mouth syndrome, stomatodynia, oral dysesthesia, pain management.

## Introduction

The patient with a complaint of a burning sensation of the oral mucosa presents one of the most difficult challenges to the health care professionals. There is a variety of names applied to this presentation including, but not limited to, burning mouth syndrome (the most widely accepted), stomatodynia, stomatopyrosis, glossopyrosis, glossodynia, sore mouth, sore tongue and oral dysesthesia. Burning mouth syndrome (BMS) is defined by the International Association for the Study of Pain as burning pain in the tongue or other oral mucous membrane associated with normal signs and laboratory findings lasting at least 4 to 6 months (1). The International Headache Society in the International Classification of Headache Disorders II classifies BMS in the category of cranial neuralgias and central causes of facial pain within the subcategory of central causes of facial pain (2). BMS is described as an intraoral burning sensation for which no medical or dental cause can be found. It is usually described as oral burning pain, sometimes with dysesthetic qualities similar to those present in other neuropathic pain conditions with the absence of clinical and laboratory abnormalities.

As a result of the variations in experienced symptoms, and despite the fact that numerous studies have been carried out, there is no universal consensus on the diagnosis, etiology and treatment of BMS. This leads to pa-tients being referred from one health care professional to another, causing an increased burden on both the health care system and the patient (3). Various groups of investigators have attempted to provide an answer to the questions regarding this topic, which is the subject of considerable controversy. The multiplicity of factors related with this nosologic entity, which in one form or another are involved in the appearance of the symptoms have made it currently one of the most debated issues (4).

## Epidemiology

The prevalence of burning mouth symptoms reported from international studies ranges from 0.7% to 4.6% (4). The considerable variation in prevalence among these studies may be because of different definitions of BMS leading to different criteria for the selection of the populations. It seems the prevalence of BMS increases with age in both males and females, with this syndrome mainly affecting females in the fifth to seventh decade (5). The mean age of BMS is between 55-60 years, with occurrence under 30 being rare (6,7). The ratio between females and males varies from 3:1 to 16:1 (8). These gender differences may be explained by biologic, psychologic, and socio-cultural factors; however, these factors are yet to be defined. It seems from these epidemiologic studies that menopausal females have a particularly high incidence of burning mouth (9). This syndrome has never been described in children or adolescents. No studies exist in relation to any occupational, educational or social grouping (10).

## Classification

There have been several proposed classification schemes to better characterize and define BMS. One of the proposed classification is based on daily fluctuations of the symptoms (6,9)

a) Type 1: Characterized by progressive pain, patients wake up without pain, which then increases throughout the day, affects approximately 35% of patients. This type may be associated with systemic diseases, such as nutritional deficiencies.

b) Type 2: Symptoms are constant throughout the day and patients find it difficult to get to sleep, represents 55%. These patients usually present associated psychological disorders.

c) Type 3: Symptoms are intermittent, with atypical location and pain. Constitutes 10% of patients. It seems that contact with oral allergens could play an important etiologic role in this group.

A more pragmatic approach is proposed by Scala et al. (4), who organize BMS into two clinical forms,‘Primary’ or Essential/ Idiopathic BMS, in which the causes cannot be identified‘Secondary’ BMS, resulting from local factors or systemic conditions.

Thus, these idiopathic and secondary criteria form two different subgroups of the same pathology.

## Etiopathogenesis

The etiology of BMS is poorly understood. Most support a multi-factorial syndrome involving the interaction of biological and psychological systems. A number of etiologies have been proposed suggesting BMS involves alterations in both central and peripheral nervous systems (11). The various factors related with the etiopathogenesis of this syndrome have been divided into local, systemic and psychological. ( Table 1)

**Table 1:**
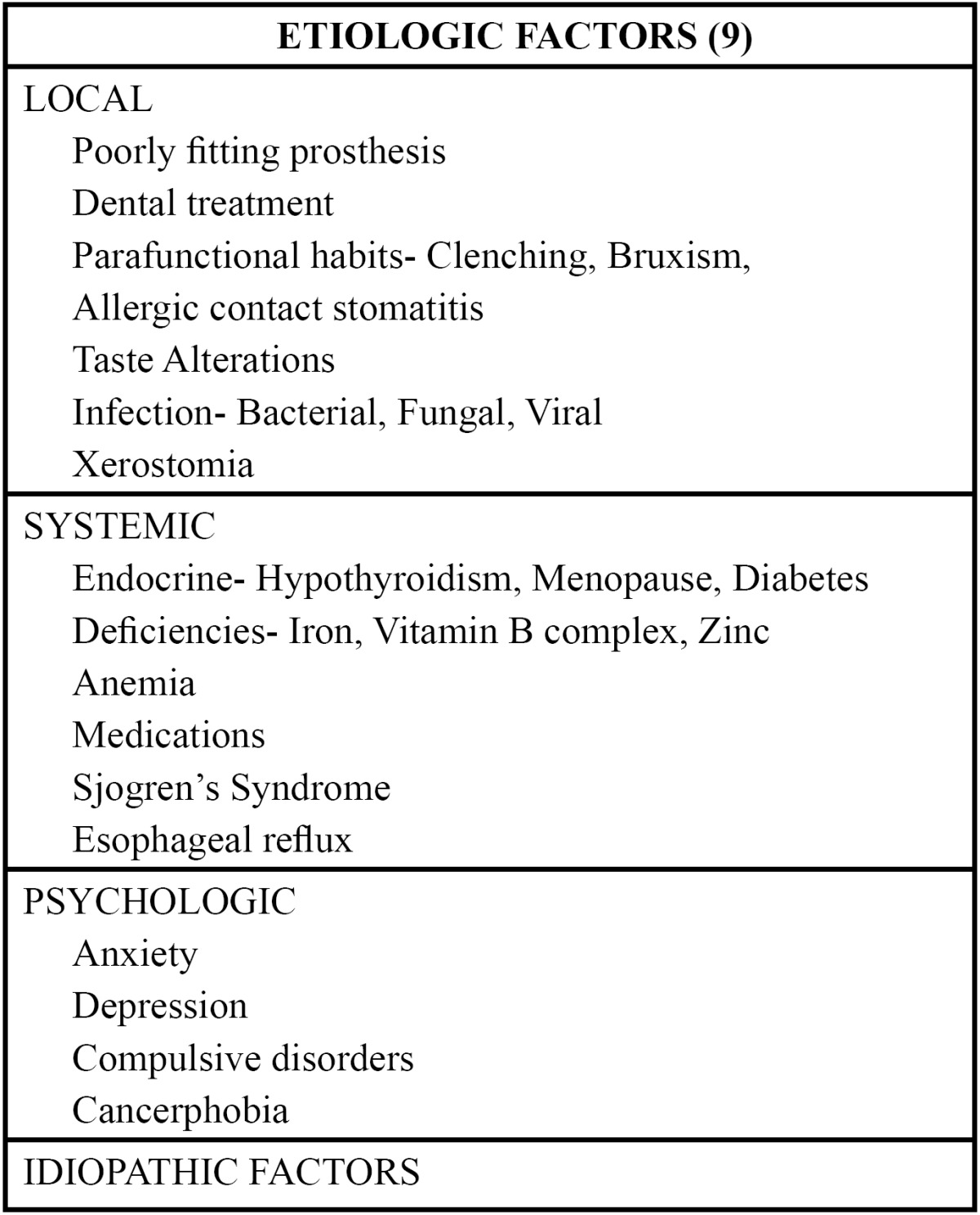
Etiologic factors for burning mouth syndrome.

A) Local Factors:

One should consider physical, chemical or biological (some bacteria or fungi) factors which have a direct irritant effect on the oral mucosa and are able to set off the burning symptoms (9). A mechanical factor to consider is the use of poorly fitting prostheses that produce microtrauma or local erythema. Local allergic reactions, due principally to high levels of residual monomers should also be considered. Infection by Candida albicans has been considered one of the most frequent factors in the production of BMS (10). Xerostomia is a concomitant symptom in patients with BMS, prevalence varying between 34 and 39% (12). In recent years investigations have been carried out into the alterations in taste perception and tolerance to pain as a possible cause of the burning sensation. This theory proposes that certain people, labeled as supertasters due to the high density of fungiform papillae present on the anterior part of the tongue, are more susceptible to developing burning mouth pain. Supertasters are principally women, and are able to perceive the bitter taste of a substance called PROP (6-n-propiltiouracilo) (4,5,9).

B) Systemic Factors:

Systemic factors implicated in BMS; many of these are deficiencies, such as vitamin deficiencies (vitamin B12, B6, C and folic acid), anemias and low levels of zinc. Hormonal changes (reduced plasma estrogens), diabetes mellitus, hypothyroidism and immunological diseases have also been described. Many medications are intimately related with burning mouth; among which are found antihistamines, neuroleptics, some antihypertensives, antiarrhythmics and benzodiazepines. Antihypertensives are among the most frequently implicated medicines, principally those that act on the renin-angiotensin system (13).

C) Psychological Factors:

Studies exist that suggest that psychopathologic factors may play an important role in BMS and support the multifactorial etiology (14,15). Many of these patients have symptoms of anxiety, depression and personality disorders, and it has been demonstrated that patients with BMS have a greater tendency towards somatization and other psychiatric symptoms (16). Cancerphobia can be present in up to 20-30% of these patients. A lower level of socialization and higher levels of somatic anxiety have been observed, as well as muscular tension, a higher tendency to worry about health and greater sadness. BMS is considered a chronic pain disorder that adversely affects quality of life (17).

Clinical Presentation

In more than one half of patients with BMS, the onset of pain is spontaneous, with no identifiable precipitating factor. Approximately one third of patients relate time of onset to a dental procedure, recent illness or medication course. Regardless of the nature of pain onset, once the oral burning starts, it often persists for prolonged period of time (18). The predominant pain character reported by BMS patients is a prolonged ‘burning’ sensation of the oral mucosa described as moderate to severe intensity that may vary throughout the course of the day (4,19). The mean severity of pain has been assessed at about 5-8cm on a 10cm visual analogue scale, where 0cm represents ‘no pain’ and 10 cm corresponds to the ‘worst possible pain’ (20) ( Table 2).

**Table 2:**
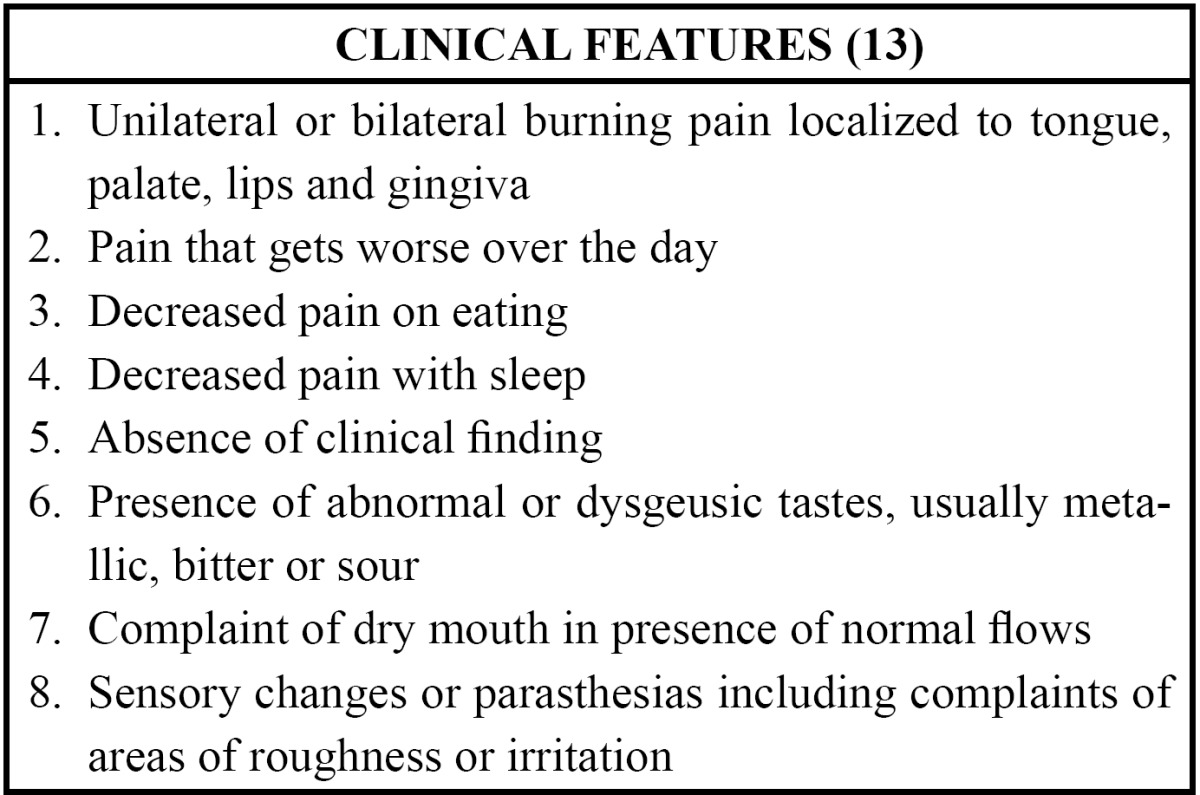
Clinical features that are helpful in the diagnosis of burning mouth syndrome.

The burning sensation often occurs in more than one oral site, with the anterior two thirds of the tongue, the anterior hard palate and the mucosa of the lower lip most frequently involved (21). Most studies have found that oral burning is frequently accompanied by other symptoms, including dry mouth and altered taste (21). Alterations in taste occur in as many as two thirds of patients and often include complaints of persistent tastes (bitter, metallic, or both) or changes in the intensity of taste perception. Damage to the taste has been reported in association with BMS, because of disinhibition of pain signaling (7). Dysgeusic tastes accompanying oral burning are often reduced by stimulation with food. Facial skin is not usually affected. In many patients with the syndrome, pain is absent during the night but occurs at a mild to moderate level by middle to late morning. Patients often report that the pain interferes with their ability to fall asleep. Perhaps because of sleep disturbances, constant pain, or both, patients with oral burning pain often have mood changes, including irritability, anxiety and depression (22). The location of oral pain is most commonly bilateral and importantly does not follow the anatomical distribution of a peripheral sensory nerve (23).

Minimal information is available on the natural course of the condition. In most cases the syndrome follows a protracted course with an average duration of 3.4years but may last for 12 years or more with recovery in up to two-thirds of patients within 6-7 years of onset (5,11). It has also been reported that BMS has a negative impact on health-related quality of life of individuals (24).

## Diagnosis

Taking a thorough and comprehensive history is the key to diagnosis of BMS. Important information to be ascertained by the practitioner relates to the past and current symptoms (pain, dry mouth, taste), their duration, intensity, character, location, onset, and factors that improve or worsen the pain and its course. A numeric or visual analog scale measuring the patient’s pain intensity and dry mouth should be used. Information should be obtained about current and past health status, including chronic systemic disorders, allergies, and immunologic disorders, and previous and current medications. This history should also include information on previous or current psychosocial stressors and psychologic well being. Important clinical characteristics that would provide a diagnosis of BMS are: a sudden or intermittent onset of pain usually localized to the tongue, hard palate, and lips; bilateral presentation; a persistent and often progressive increase in pain during the day often not present on awakening and the remission of pain with eating and sleeping; subjective sensations of a dry mouth and intraoral areas of roughness, irritation, or swelling and parafunctional habits (14).

The clinical examination is more to rule out any possible local factors that may be responsible for the oral bur-ning complaints. The clinical examination should include an extra-oral and intraoral examination of temporo-mandibular joint function; inspection and palpation of the masticatory muscles, oral mucosa, tongue mobility, and dental hard and soft tissues; and evaluation of any prosthetic devices. Objective measurements of salivary flow rates (whole stimulated and unstimulated saliva) and taste function should be taken (25). Neurologic imaging and consultation should be a consideration if patients present with more complex, confounding, or atypical symptoms, including sensory, motor, and autonomic changes, to rule out any neurodegenerative disorders or central nervous system pathology (9) ( Table 3).

**Table 3:**
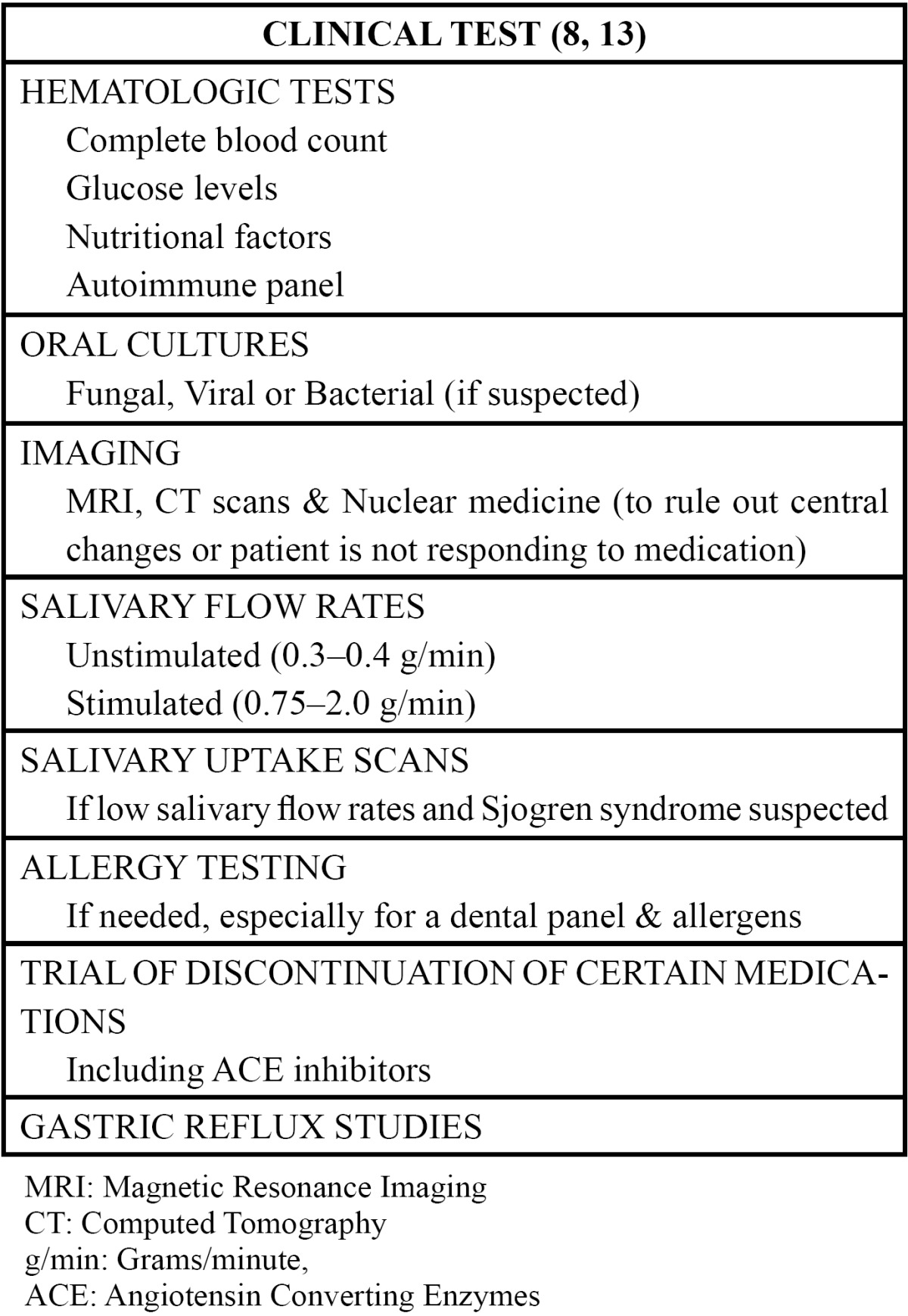
Clinical tests for burning mouth syndrome.

The diagnosis is usually late, often due to a lack of understanding of the nature of this entity, in addition to the patients taking up many health resources, since they frequently consult various specialists. It is important to highlight that the diagnosis of BMS should be established only when all other possible causes have been discounted, being a diagnosis by elimination (10).

## Treatment

The management of BMS has been quite disappointing to date – this in part being due to our lack of knowledge of the specific mechanisms underlying the syndrome. The symptoms of BMS tend to become chronic. This complicates patient management and gives rise to situations similar to those found in chronic pain, where symptoms persistence over time gives rise to increased anxiety and depression. Paramount to the clinical management of BMS is obtaining the correct diagnosis. Also, it has been proved that the sooner treatment is prescribed after the diagnosis of burning mouth syndrome (BMS), the better the results obtained (26).

Initially, the clinician must determine if the patient is suffering from primary BMS or secondary BMS (27). Secondary BMS requires appropriate diagnosis and treatment of the underlying condition to manage symptoms. In primary BMS the cause is unclear, so treatment options are based on patients’ symptomatology. Three approaches or combinations that can be considered part of the management strategy include topical medications, systemic medications and behavioral interventions (9) ( Table 4).

**Table 4:**
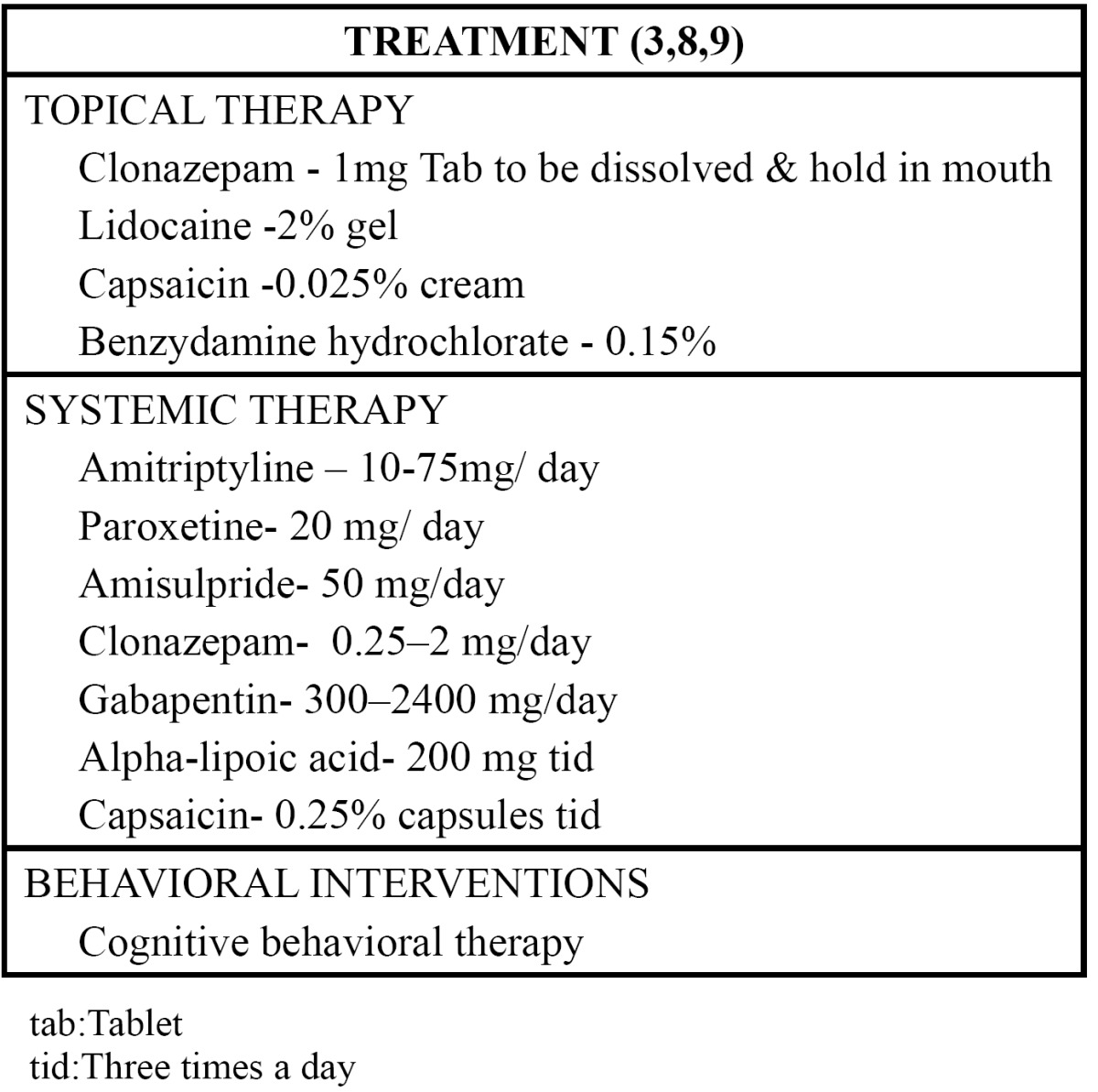
Treatment of burning mouth syndrome.

The most-used medications to treat this syndrome are antidepressants, antipsychotics, antiepileptics, analgesics and oral mucosa protectors. The tricyclic antidepressants such as amitriptyline and nortriptyline at low doses are useful in BMS, although some authors contraindicate their use in patients with dry mouth as they can worsen the condition (10). Studies have been made to evaluate the efficacy and tolerance of amisulpiride (50 mg/day) and selective serotonin inhibitors: paroxetine (20 mg/day) and sertraline (50 mg/day) in the treatment of BMS, over eight weeks, with a reasonably high efficacy (around 70%) (28). The efficacy of oral clonazepam (0.25 mg/day increasing increasing to a maximum of 3 mg/day) has also been evaluated with variable results, or by topical application (0.5 mg to 1 mg two or three times a day) with better results (29).

Gabapentin has demonstrated mixed results and studies are ongoing with pregabalin. The medication is administered at an initial dose of 300 mg/day, increasing by 300 mg/day every two days to a maximum of 2,400 mg/day (30,31). Topical capsaicin has also been applied in BMS, used as a desensitizing agent in patients with BMS, but it is usually unaccepted by patients due to its taste. Topical capsaicin has been used as a treatment alternative for controlling neuropathic pain in general. The drug is normally used at concentrations of between 0.025% and 0.075%, inducing desensitization to thermal, chemical and mechanical stimuli when applied topically. However, it should be noted that there are clear limita¬tions to the use of topical capsaicin, such as limited effect over time and a limited magnitude of improvement (32). Systemic capsaicin has been used (0.25%, three times a day, for 30 days) with a significant reduction in pain intensity compared with a placebo group (33).

Alpha lipoic acid is a powerful neuroprotector that prevents damage to nerve cells by free radicals. It significantly reduces the symptoms in the majority of patients with idiopathic dysgeusia. Several studies suggest that alpha lipoic acid can improve the symptoms in BMS, showing that at two months, 97% of the patients treated with alpha lipoic acid (200 mg, three times a day) experienced an improvement in the symptoms (34).

Topical steroid hormones and anti-inflammatory rinses have been tried with little evidence of effectiveness in reducing or eliminating the symptoms of burning mouth syndrome, particularly when compared to placebo or spontaneous remission rates (35).

Hormone replacement therapy (HRT) has also been used, finding that women with symptoms of burning and estrogen receptors in the oral mucosa respond to hormone replacement, while this does not occur in patients without these receptors; however it cannot be guaranteed that HRT could be an effective treatment for the oral symptomatology (10).

Studies on the effect of cognitive therapy on resistant BMS shows a statistically significant reduction in pain intensity for those receiving cognitive therapy compared with placebo immediately following the therapy and a further reduction at the 6-month follow up (36). Another study showed some improvement of BMS resulting from psychotherapy treatment over 2 months, with significant improvement when combined with alpha-lipoic acid therapy (ALA) (600 mg/d) (37). It seems from these studies that the practitioner may consider the involvement of a behavioral medicine practitioner as part of a multidisciplinary approach when managing patients who have BMS.

## Conclusions

Burning Mouth Syndrome remains a fascinating, though poorly understood, condition in the field of oral medicine. No consensus exists in defining, diagnosing and treating BMS. Furthermore, the lack of understanding the cause and mechanism behind the syndrome adds to the difficulty in finding a therapeutic management program. There is little evidence-based material to assist the practitioner when dealing with these individuals. There is no doubt that innovative and interdisciplinary research is required to elucidate and expand on the knowledge of the etiology and pathogenic factors involved in BMS. The positive aspect is that most patients can be helped and many achieve a complete cure of their condition. It relies, however, on initial recognition and this is the most critical step.
